# Review of the millipede genus *Kronopolites* Attems, 1914 (Diplopoda, Polydesmida, Paradoxosomatidae), with the description of a new species from Laos

**DOI:** 10.3897/zookeys.472.9001

**Published:** 2015-01-19

**Authors:** Natdanai Likhitrakarn, Sergei I. Golovatch, Somsak Panha

**Affiliations:** 1 Division of Plant Protection, Faculty of Agricultural Production, Maejo University, Chiang Mai, 50290, Thailand; 2 Institute for Problems of Ecology and Evolution, Russian Academy of Sciences, Leninsky pr. 33, Moscow 119071, Russia; 3 Animal Systematics Research Unit, Department of Biology, Faculty of Science, Chulalongkorn University, Bangkok, 10330, Thailand

**Keywords:** Millipede, *Kronopolites*, new species, key, distribution, Paradoxosomatidae

## Abstract

The millipede genus *Kronopolites* currently comprises 11 species, including a new species from northern Laos: *Kronopolites lunatus* **sp. n.** The generic diagnosis is updated, a key given to all known species, and their distributions are mapped.

## Introduction

The flat-back millipede genus *Kronopolites* Attems, 1914 is widespread in tropical Asia ranging from the Himalayas of Kashmir, India in the west to Taiwan in the east (Fig. 4). This genus belongs to the mainly Southeast Asian tribe Sulciferini in the family Paradoxosomatidae which is one of the largest families in the entire class Diplopoda, dominating the millipede fauna of Indo-Australia (Jeekel 1968, Nguyen and Sierwald 2013). The genus *Kronopolites* currently contains 10 described species (Golovatch 2013a, 2013b, 2014): *Kronopolites swinhoei* (Pocock, 1895), the type-species which is widespread in central and southeastern China, *Kronopolites acuminatus* Attems, 1937, from northern Vietnam, *Kronopolites formosanus* (Verhoeff, 1939), from northern Taiwan, *Kronopolites biagrilectus* Hoffman, 1963, from Jiangxi Province, China, *Kronopolites fuscocingulatus* Jeekel, 1982, from northern Thailand, *Kronopolites occidentalis* Golovatch, 1983, from the Kashmir Himalaya, India, *Kronopolites montanus* Golovatch, 2009, from northern Vietnam, *Kronopolites rugosus* Golovatch, 2013 and *Kronopolites davidiani* Golovatch, 2014, both from Yunnan Province, China, as well as *Kronopolites semirugosus* Golovatch, 2013 from Sichuan Province, China.

The present study treats some new material collected in Laos during several field trips. Prompted by the discovery of a new species, the authors have revised the entire genus *Kronopolites* adding a new diagnosis and updating both the catalogue and key to species. In addition, its distribution is mapped.

## Material and methods

Material was collected in northern Laos in 2014 by SP and members of the Animal Systematics Research Unit, Chulalongkorn University. Specimens were preserved in 75% ethanol, and morphological investigations were carried out in the laboratory using an Olympus stereomicroscope. Scanning electron micrographs (SEM) of gonopods coated with gold were taken using a SEM JEOL JSM–5410 LV microscope. The gonopods were then removed from stubs and returned to alcohol after examination. Digital images of freshly fixed specimens were taken in the laboratory and assembled using the “Cell^D^” automontage software of the Olympus Soft Imaging Solution package. In addition, line drawings of gonopod characters were also prepared. The types are housed in the Museum of Zoology, Chulalongkorn University (CUMZ), Bangkok, Thailand.

Collecting sites were located by GPS using the WGS84 datum.

In the catalogue sections, D stands for the original description, subsequent descriptive notes or appearance in a key, R for a subsequent record or records, and M for a mere mention.

## Taxonomic part

### Family Paradoxosomatidae Daday, 1889 Subfamily Paradoxosomatinae Daday, 1889 Tribe Sulciferini Attems, 1898

#### Genus Kronopolites

Taxon classificationAnimaliaPolydesmidaParadoxosomatidae

Attems, 1914

Kronopolites Attems 1914: 219 (D).
Kronopolites – Attems 1929: 272 (D); 1931: 113 (D); 1936: 225 (D); 1937: 49 (D); Verhoeff 1939: 274 (D); Takashima 1950: 38 (M); Takakuwa 1954: 30 (D); Hoffman 1963: 579 (D); 1980: 169 (M); Jeekel 1968: 71 (R); 1971: 225 (M); 1982: 243 (M); 1988: 98 (M); Chen et al. 2006: 252 (M); Golovatch 2009: 121 (D); 2013a: 12 (M); Nguyen and Sierwald 2013: 1287 (M).
Kansupus Verhoeff 1934: 17 (D), synonymized by Attems (1936: 233).
Kansupus – Jeekel 1971: 225 (M); Hoffman 1980: 169 (M).
Parakansupus Verhoeff 1939: 273 (D), synonymized by Hoffman (1963: 579).
Parakansupus – Jeekel 1971: 230 (M); Hoffman 1980: 169 (M).

##### Diagnosis.

Body medium-sized to large (ca 23–42 mm long, ca 1.6–6.5 mm wide), with 20 segments. Paraterga from poorly to strongly developed, mostly without lateral incisions. Transverse metatergal sulcus distinct. Sterna usually modified, an acute cone often present near each coxa. Sternal lobe or cone(s) between ♂ coxae 4 present or absent. Pleurosternal carinae usually well-developed.

Gonopods rather simple to relatively complex; coxites elongate, subcylindrical, distoventrally sparsely setose, without tubercles; prefemoral (= setose) part of telopodite moderate to relatively large, 1/3–1/2 as long as acropodite; femorite rather slender to stout, slightly curved, enlarged distad, with an evident groove on mesal face and a distinct distolateral sulcus demarcating a postfemoral part; the latter typically carrying a fork consisting of two lateral/ventral processes: usually a smaller basal process **b** with its tip pointed basad to prefemoral part, and a larger, normally suberect or ventrally curved process **a**; solenophore strongly developed, slender, slightly longer than or nearly as long as femorite, strongly curved mesad, sometimes with a membranous, distally strongly expanded end, almost completely sheathing a flagelliform and longer solenomere; seminal groove running entirely or mostly mesally along an excavate femorite, then directed slightly dorsad in distal part of femorite to follow onto solenomere thereafter.

##### Type species.

*Strongylosoma swinhoei* Pocock, 1895, by original designation.

##### Other species included.

*Kronopolites acuminatus* Attems, 1937, *Kronopolites formosanus* (Verhoeff, 1939), *Kronopolites biagrilectus* Hoffman, 1963, *Kronopolites fuscocingulatus* Jeekel, 1982, *Kronopolites occidentalis* Golovatch, 1983, *Kronopolites montanus* Golovatch, 2009, *Kronopolites rugosus* Golovatch, 2013, *Kronopolites semirugosus* Golovatch, 2013, *Kronopolites davidiani* Golovatch, 2014, *Kronopolites lunatus* sp. n.

##### Remarks.

Pocock (1895) described the type species in *Strongylosoma* Brandt, 1833, from a single female from Chee Foo, China. Soon after that Brölemann (1896), having received a male of this species from Chou-San Island, China, gave a more detailed description, including that of gonopod structure. Attems (1914) proposed a new genus, *Kronopolites*, and designated *Strongylosoma swinhoei* as type species.

#### Kronopolites acuminatus

Taxon classificationAnimaliaPolydesmidaParadoxosomatidae

Attems, 1937

Kronopolites acuminatus Attems 1937: 52 (D).
Kronopolites acuminatus – Attems 1938: 227 (D); Jeekel 1968: 59 (R); Enghoff et al. 2004: 38 (M, R); Golovatch 2009: 121 (D); Nguyen and Sierwald 2013: 1287 (M).
Kronopolites acuminatus acuminatus – Hoffman 1963: 584 (M, R); Golovatch 1983a: 181 (M); Enghoff et al. 2004: 38 (M, R).

##### Remarks.

This species was described from Hagiang, Hagiang Province, Vietnam (Attems 1937), later redescribed from the type locality (referred to as Ha Giang, 22°50'N, 105°E, 20 miles south of the Vietnam-China frontier) (cf. Hoffman 1963).

#### Kronopolites biagrilectus

Taxon classificationAnimaliaPolydesmidaParadoxosomatidae

Hoffman, 1963

Kronopolites acuminatus biagrilectus Hoffman 1963: 584 (D).
Kronopolites acuminatus biagrilectus – Wang and Mauriès 1996: 86 (M); Enghoff et al. 2004: 38 (M); Jeekel 1968: 71 (M); Sierwald 2009: 125 (M).
Kronopolites biagrilectus – Golovatch 2009: 121 (D); Nguyen and Sierwald 2013: 1287 (M).

##### Remarks.

This species was described from Kuling, 29°30'N, 116°E, 10 miles south of Kiukiang, Kiangsi (= Guangxi) Province, China (Hoffman 1963).

#### Kronopolites davidiani

Taxon classificationAnimaliaPolydesmidaParadoxosomatidae

Golovatch, 2014

Kronopolites davidiani Golovatch 2014: 10 (D).

##### Remarks.

This species has been described from near Wenchian, 3365 m a.s.l., 27°20'35"N, 99°52'34"E, 214 National Road, Yunnan (not Sichuan!) Province, China (cf. Golovatch 2014).

#### Kronopolites formosanus

Taxon classificationAnimaliaPolydesmidaParadoxosomatidae

(Verhoeff, 1939)

Kronopolites (Parakansupus) formosanus Verhoeff 1939: 273 (D).
Kronopolites formosanus – Attems 1940: 540 (D); Takashima 1950: 38 (R); Chamberlin and Wang 1953: 5 (R); Wang 1964: 69 (M); Takakuwa 1954: 31 (D); Hoffman 1963: 585 (D); Jeekel 1968: 71 (M); Golovatch 1983b: 298 (M); 2009: 121 (D); Chen et al. 2006: 259 (D); Nguyen and Sierwald 2013: 1287 (M).
Kronopolites ralphi Wang 1957: 106 (D), synonymized by Hoffman (1963: 585).
Kronopolites ralphi – Wang 1958: 342 (R); 1964: 69 (M).

##### Remarks.

This species had been erroneously listed as a synonym of *Kronopolites swinhoei* by Wang and Mauriès (1996: 86) and by Korsós (2004: 23) until these mistakes were corrected by Chen et al. (2006). In fact, *Kronopolites formosanus* is endemic to northern Taiwan (Verhoeff 1939, Chamberlin and Wang 1953, Wang 1957), occurring below 1000 m a.s.l.: FuShan Botanical Garden, 726 m a.s.l., Ulai, Taipei County; Yang Ming Shan National Park, ca. 750 m a.s.l., near YuYouRen Tomb, Taipei City, Taiwan (Chen et al. 2006).

#### Kronopolites fuscocingulatus

Taxon classificationAnimaliaPolydesmidaParadoxosomatidae

Jeekel, 1982

Kronopolites fuscocingulatus Jeekel 1982 (D): 238 (D).
Kronopolites fuscocingulatus – Enghoff 2005: 97 (R); Golovatch 2009: 121 (D); Nguyen and Sierwald 2013: 1288 (M).

##### Remarks.

Jeekel (1982) described this species from several places in northern Thailand: Hakka village, 50 km N of Chiang Rai City, 800–900 m a.s.l.; Mac Chan (= Mae Chan), Mae Chan District, Chiang Rai Province; Doi Suthep National Park, Chiang Mai Province. Later, Enghoff (2005) reported new specimens of this species in his checklist: Doi Pha Hom Pok National Park, Northwest of Fang, 1550–1660 m a.s.l.; limestone area, 1300 m a.s.l., Doi Chiang Dao National Park; Kontathan (= Montha Than) Waterfall area, Doi Suthep National Park, Chiang Mai Province.

#### Kronopolites montanus

Taxon classificationAnimaliaPolydesmidaParadoxosomatidae

Golovatch, 2009

Kronopolites montanus Golovatch 2009: 121 (D).
Kronopolites montanus – Nguyen and Sierwald 2013: 1288 (M).

##### Remarks.

This species was described from Hoang Lien National Park, ca 2000 m a.s.l., west of Sapa, Lao Cai Province, Vietnam (Golovatch 2009).

#### Kronopolites occidentalis

Taxon classificationAnimaliaPolydesmidaParadoxosomatidae

Golovatch, 1983

Kronopolites occidentalis Golovatch 1983b: 297 (D).
Kronopolites occidentalis – Golovatch 1984: 328 (R); 2009: 121 (D); Nguyen and Sierwald 2013: 1288 (M); Shelley 2014: 3 (R).

##### Remarks.

This species was described from Pir Panjal Mountains, 2600 m a.s.l., Tangmarg, Jammu and Kashmir State, India (Golovatch 1983). New specimens were collected near the ruins of Pari Mahal Monastery, 1500 m a.s.l., Srinagar, Jammu and Kashmir State, India (Golovatch 1984).

#### Kronopolites rugosus

Taxon classificationAnimaliaPolydesmidaParadoxosomatidae

Golovatch, 2013

Kronopolites rugosus Golovatch 2013a: 12 (D).
Kronopolites rugosus – Nguyen and Sierwald 2013: 1288 (M); Golovatch 2013b: 311 (M).

##### Remarks.

This species has been described from north of Lijiang, 27°01'N, 100°12'E, 2400 m a.s.l., Yunnan Province, China (Golovatch 2013a).

#### Kronopolites semirugosus

Taxon classificationAnimaliaPolydesmidaParadoxosomatidae

Golovatch, 2013

Kronopolites semirugosus Golovatch 2013b: 311 (D).

##### Remarks.

This species was described from NW of Mianning, 2955 m a.s.l., 28°39'13"N, 101°58'34"E, Sichuan Province, China (Golovatch 2013b).

#### Kronopolites swinhoei

Taxon classificationAnimaliaPolydesmidaParadoxosomatidae

(Pocock, 1895)

Stronglosoma Swinhoei Pocock 1895: 354 (D).
Kronopolites Swinhoei – Brölemann 1896: 354 (D); Attems 1898: 304 (D); 1914: 219 (R).
Kronopolites swinhoei – Attems 1936: 226 (D); 1937: 51 (D); Chamberlin and Wang 1953: 5 (R); Hoffman 1963: 581 (D); Jeekel 1968: 71 (M); Golovatch 1978: 678 (R); 1983b: 298 (M); 2009: 121 (D); 2013a: 2 (R, M); Wang and Mauriès 1996: 86 (M); Geoffroy and Golovatch 2004: 20 (R); Korsós 2004: 23 (R, M); Chen et al. 2006: 252 (M); Nguyen and Sierwald 2013: 1286 (M).
Kronopolites swinhoei swinhoei – Attems 1937: 51 (D).
Kansupus svenhedini Verhoeff 1934: 17 (D), synonymized by Hoffman (1963: 581).
Kronopolites svenhedini – Attems 1936: 233 (R); 1937: 53 (D); Zhang and Li 1978: 12 (R); Wang and Mauriès 1996: 86 (M).
Kansupus svenhedini var. dentiger Verhoeff 1934: 19 (D), synonymized by Hoffman (1963: 581).
Kronopolites svenhedini dentiger – Attems 1936: 233 (R); 1937: 54 (D).

##### Remarks.

This species is especially widely distributed in mainland China: Chee Foo (Pocock 1895); Chou San Island (Brölemann 1896); Lan Tschou, Gansu (Attems 1936); Pei-shui-ho, 700 m a.s.l., northeastern Sichuan and southern Gansu (Attems 1937); Wenchow (= Yung-chia), Chekiang Province; Chekiang Province (Chamberlin and Wang 1953), Hangchow, Chekiang Province (Hoffman 1963); Taibai Shan Mountains, 1300–1700 m a.s.l.; southern slopes, above Houshenzi, 33°51'N, 107°50'E, Shaanxi Province; Bei Shan National Park, 36°56'N, 102°39'E, ca 90 km NE of Xining, Gansu (not Qinghai) Province (corrected here versus Golovatch 2013a); Grotte du Cirque (Circus Cave), Zheng Xiong County, Yunnan Province; Cave Yan Bao Dong, Zheng Xiong County, Yunnan Province; Cave Ha Chong Dong, near Xingren Huawu, Guizhou Qianxi Province, China (Geoffroy and Golovatch 2004).

#### Kronopolites lunatus sp. n.

Taxon classificationAnimaliaPolydesmidaParadoxosomatidae

http://zoobank.org/BCEF7CDD-7BE6-4B47-B02D-5FA3F658A242

Figs 1 , 2 , 3

##### Holotype

♂, Laos, Xieng Khouang Province, Phookood District, Cave Pra, ca 1180 m a.s.l., 19°30'02"N, 102°52'20"E, 02.07.2014, leg. R. Srisonchai.

##### Paratype.

1 ♂, Laos, Luang Prabang Province, Chomphet District, Kacham Waterfall, ca 440 m a.s.l., 19°38'57"N, 102°04'52"E, 01.07.2014, leg. C. Sutcharit.

##### Name.

To emphasize the lateral crescent-shaped processes on the gonopod.

##### Diagnosis.

Superficially very similar to *Kronopolites acuminatus*, but differs in the smaller size, the width of midbody pro- and metazonae being 2.4–2.5 and 3.1–3.2 mm, respectively (versus 4.5 mm and 6.5 mm, respectively); tarsal brushes are present until ♂ leg 9 (versus absent), and gonopod process **b** is > 2 times as long as process **a** (versus shorter), process **a** being clearly curved (versus nearly straight) while process **b** is enlarged and lies adjacent to the femorite (versus clearly separated from the femorite). Eventually, it keys out closest to *Kronopolites formosanus* (see Key below).

##### Description.

Length 28.4–29.5 (♂), width of midbody pro- and metazonae 2.4–2.5 and 3.1–3.2 mm (♂), respectively.

**Figure 1.**
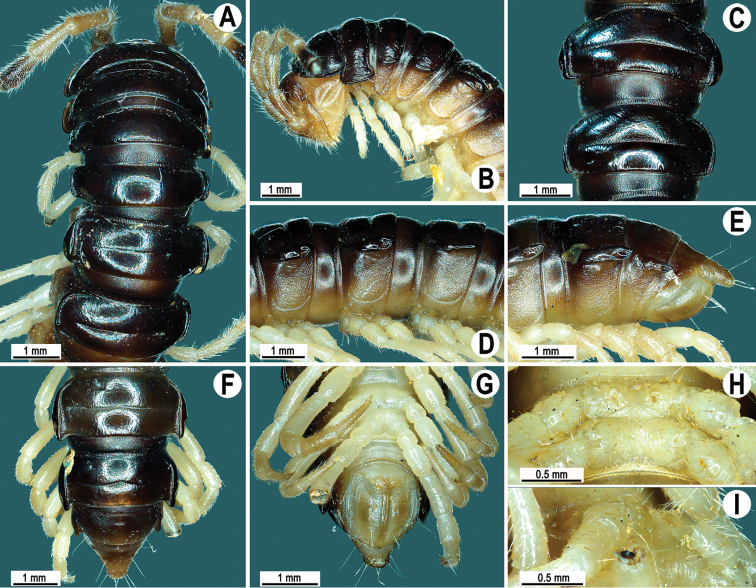
*Kronopolites lunatus* sp. n., ♂ paratype. **A, B** anterior part of body, dorsal and lateral views, respectively **C** segments 10 and 11, dorsal view **D** segments 9–11, lateral view **E–G** posterior part of body, lateral, dorsal and ventral views, respectively **H, I** sternal cones between coxae 4, subcaudal and sublateral views, respectively.

Live coloration mostly dark, blackish brown; antennae and head dark brown to light brown, venter and a few basal podomeres light brown to yellow-brown; coloration of alcohol material after four months of preservation faded to dark brown; antennae and epiproct light brown to light yellow, venter and a few basal podomeres light brown to pallid (Fig. 1A–I).

Clypeolabral region and vertex densely setose, epicranial suture distinct. Antennae moderately long (Fig. 1A), extending behind body segment 3 (♂) when stretched dorsally. In width, segment 4 < 3 < head < 5 < collum < segment 2 < 6–17 (♂); thereafter body gently and gradually tapering. Collum with three transverse rows of setae: 4+4 anterior, 3+3 intermediate and 4+4 posterior; lateral incisions absent; caudal corner of paraterga very broadly rounded, declined ventrad, produced behind rear tergal margin (Fig. 1A, B).

Tegument smooth and shining, prozonae finely shagreened, metaterga finely rugulose (Fig. 1A, C, F); surface below paraterga finely microgranulate (Fig. 1B, D, E). Postcollum metaterga with two transverse rows of setae: 3+3 in anterior (pre-sulcus) and 3+3 in posterior (post-sulcus) row, traceable as insertion points. Tergal setae long and slender, mostly abraded, about 1/3 as long as metaterga. Axial line barely traceable both on pro- and metazonae. Paraterga strongly developed (Fig. 1A–F), lying rather high (at upper 1/3 of body), slightly upturned, but lying below dorsum; anterior edge broadly rounded and narrowly bordered, fused to callus; caudal corner very narrowly rounded, starting from segment 15 extending increasingly well beyond rear tergal margin (Fig. 1E, F); lateral edge without incisions (Fig. 1A, C, F); posterior edge nearly straight. Calluses on paraterga narrow, delimited by a sulcus both dorsally and ventrally. Ozopores evident, lateral, lying in an ovoid groove at about 1/4 in front of posterior edge of metaterga. Transverse sulcus usually distinct (Fig. 1A, C, F), slightly incomplete on segment 19, complete on metaterga 3–18 (♂), narrow, line-shaped, shallow, reaching bases of paraterga, faintly ribbed at bottom. Stricture between pro- and metazonae evident, broad and deep, ribbed at bottom down to base of paraterga (Fig. 1A–F). Pleurosternal carinae complete crests with a sharp caudal tooth on segments 2–7, thereafter increasingly strongly reduced until segment 17 (♂). Epiproct (Fig. 1E–G) conical, flattened dorsoventrally, with two small apical papillae; tip subtruncate; pre-apical papillae small, lying close to tip. Hypoproct roundly subtriangular, setiferous knobs at caudal edge small and well-separated (Fig. 1G).

Sterna densely setose, without modifications, but with two small, rounded, fully separated, setose cones between ♂ coxae 4 (Fig. 1H, I). Legs rather long and slender, midbody ones ca 1.2–1.3 (♂) as long as body height (Fig. 1A, B, F, G); prefemora without modifications, tarsal brushes present until ♂ leg 9.

**Figure 2.**
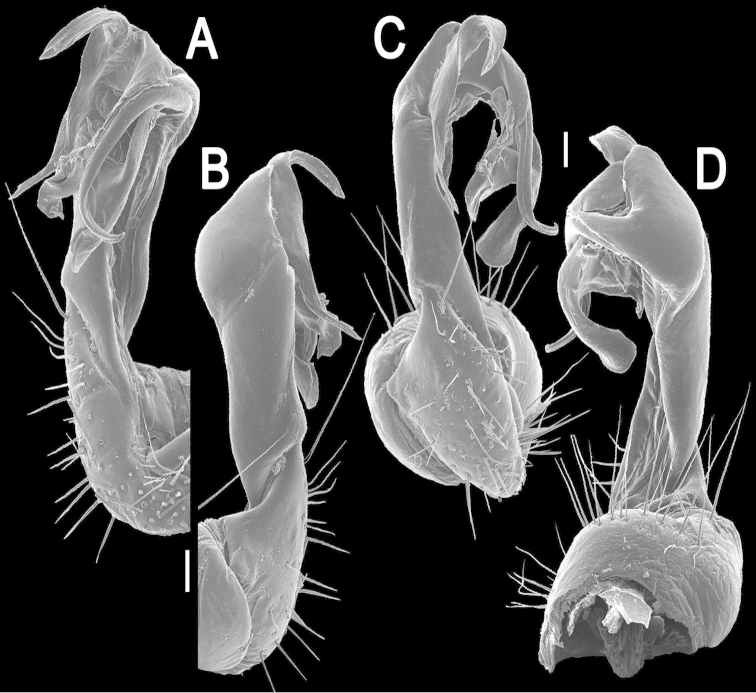
*Kronopolites lunatus* sp. n., ♂ holotype, right gonopod. **A–D** mesal, lateral, subcaudal and suboral views, respectively. Scale bars: 0.1 mm.

**Figure 3.**
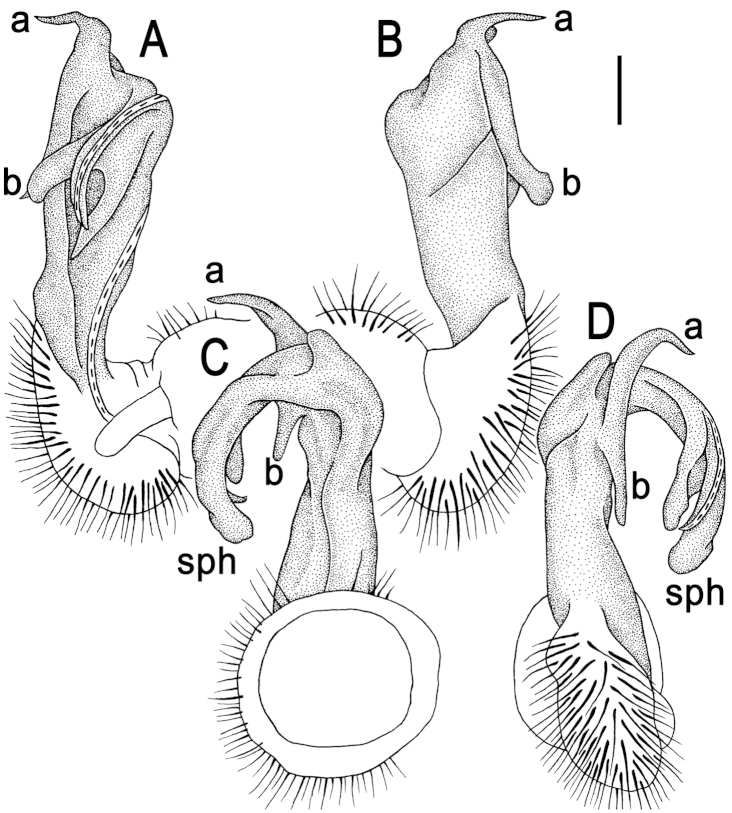
*Kronopolites lunatus* sp. n., ♂ holotype, right gonopod. **A–D** right gonopod, mesal, lateral, oral and caudal views, respectively. Scale bar: 0.2 mm.

Gonopods (Figs 2, 3) rather complex; coxa a little curved caudad, sparsely setose distoventrally. Prefemur densely setose, about 1/3 as long as femorite + postfemoral part. Femorite rather stout, with an evident mesal groove and a strong distolateral sulcus demarcating a postfemoral part; the latter well-developed, with very prominent, bipartite, crescent-shape, lateral processes: process **a** rather short, coiled and pointed; process **b** long and coiled, also pointed; solenophore clearly curved, long, expanded distomesally, trifid, lamina medialis supporting a long flagelliform solenomere.

##### Remarks.

This is the first *Kronopolites* to be found in Laos.

### Key to the species of *Kronopolites*, chiefly based on ♂ characters (modified after Golovatch 2009)

**Table:** 

1	Coloration with a contrasting pattern, some parts of body segments being much paler, some other ones much darker	**2**
–	Coloration rather uniformly brown to brown-blackish, only venter and legs largely yellowish (Fig. 1A–I)	**8**
2	Paraterga relatively poorly developed, set low (mostly at about upper 1/3 of segments), caudal corners of midbody paraterga usually not projecting behind rear tergal margin, at most narrowly rounded (Fig. 1C, D)	**3**
–	Paraterga usually relatively well developed, mostly set higher, caudal corners of midbody paraterga produced behind rear tergal margin, acuminate	**6**
3	Sternal cones on ♂ coxae 4 missing; processes **a** and **b** of gonopod nearly independent, slender and long. Northern Thailand	***Kronopolites fuscocingulatus***
–	Sternal cones on ♂ coxae 4 present, processes **a** and **b** of gonopod on a broad common stem, shorter. China	**4**
4	Surface of metaterga rather smooth; gonopod femorite slender, process **a** longer, process **b** shorter, beak-shaped	***Kronopolites swinhoei***
–	Surface of metaterga rugose; gonopod femorite stout, processes **a** and **b** of gonopod different	**5**
5	Process **a** of gonopod short and spiniform, process **b** large and axe-shaped	***Kronopolites rugosus***
–	Processes **a** and **b** of gonopod subequal in length, ribbon-shaped	***Kronopolites semirugosus***
6	Coloration dark brown with yellow paraterga; sternal cones between ♂ coxae 4 missing; processes **a** and **b** of gonopod short and small, sharing a very distinct common stem; Kashmir Himalayas	***Kronopolites occidentalis***
–	Colour pattern different, rear halves of prozonae and fore halves of metazonae usually being black-brown, remaining parts yellowish; sternal cones between ♂ coxae 4 present; processes **a** and **b** of gonopod longer and slenderer, their shared base far less conspicuous	**7**
7	Process **a** of gonopod somewhat shorter than process **b.** Northern Vietnam	***Kronopolites acuminatus***
–	Process **a** of gonopod somewhat longer than process **b.** Jiangxi Province, China	***Kronopolites biagrilectus***
8	Paraterga relatively well developed (Fig. 1A, C, F); pleurosternal carinae evident in ♂ segments 2–16; process **a** of gonopod clearly shorter than **b**	**9**
–	Paraterga rather poorly developed; pleurosternal carinae evident until ♂ segment 10 at most; processes **a** and **b** of gonopod subequal in length	**10**
9	Sternal cones between ♂ coxae 4 present; ♂ tarsal brushes missing; solenophore with conspicuous bipartite, complex, apical processes	***Kronopolites montanus***
–	Sternal cones on ♂ coxae 4 missing; ♂ tarsal brushes present until legs of segment 17; solenophore simple and slender, with a little branch set off before apex	***Kronopolites davidiani***
10	Sternal cone between ♂ coxae 4 single, large. Northern Taiwan	***Kronopolites formosanus***
–	Two small sternal cones between ♂ coxae 4 (Fig. 1H, I). Northern Laos	***Kronopolites lunatus* sp. n.**

## Conclusions

**Figure 4.**
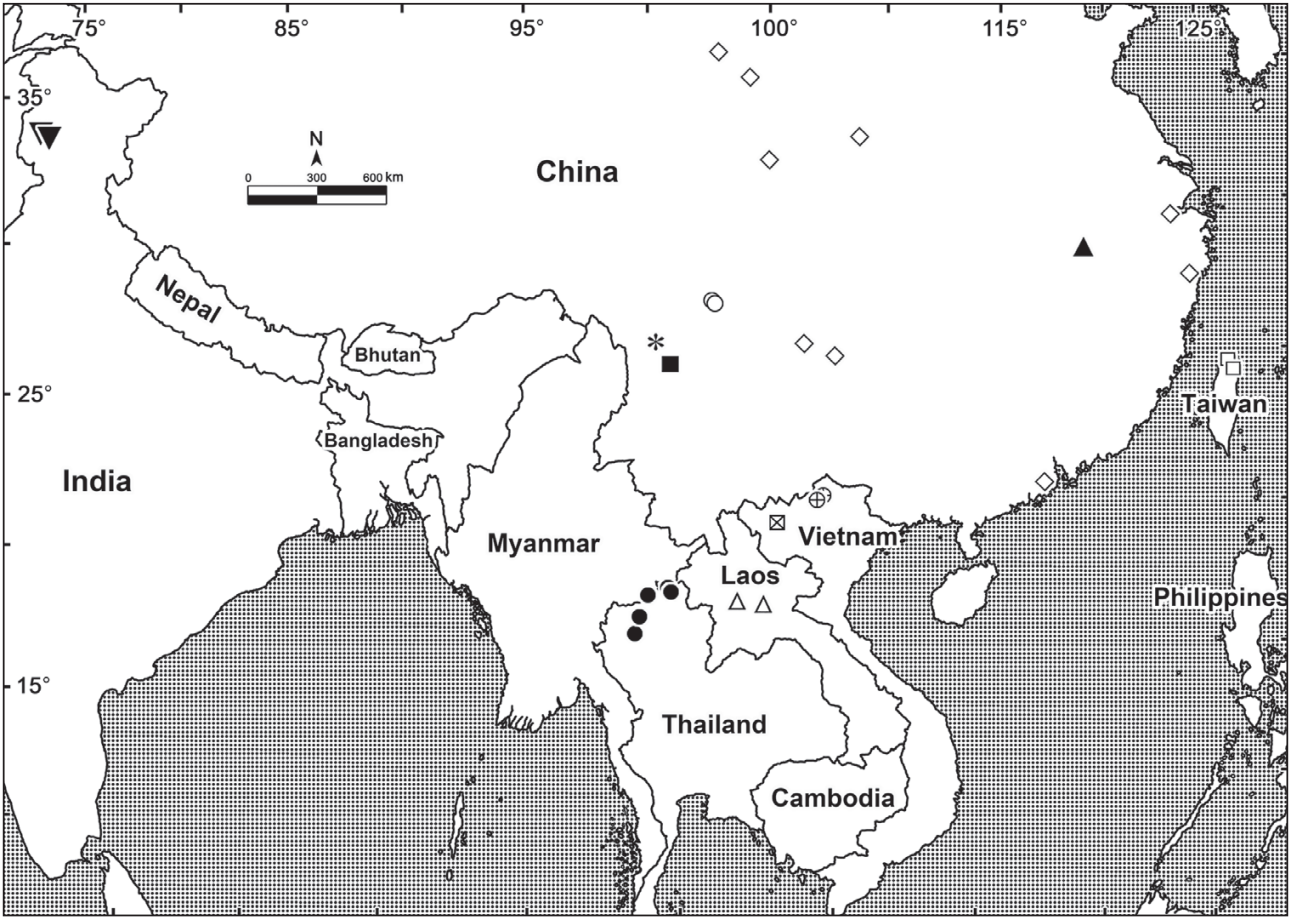
Distribution of *Kronopolites* (11 species). Inverted filled triangle, more or less from west to east: *Kronopolites occidentalis* Golovatch, 1983; Filled circle: *Kronopolites fuscocingulatus* Jeekel, 1982; Open circle: *Kronopolites semirugosus* Golovatch, 2013; Asterisk: *Kronopolites davidiani* Golovatch, 2014; Filled square: *Kronopolites rugosus* Golovatch, 2013; Open triangle: *Kronopolites lunatus* sp. n.; Crossed square: *Kronopolites montanus* Golovatch, 2009; Cross circle: *Kronopolites acuminatus* Attems, 1937; Open diamond: *Kronopolites swinhoei* (Pocock, 1895); Filled triangle: *Kronopolites biagrilectus* Hoffman, 1963; Open square: *Kronopolites formosanus* (Verhoeff, 1939).

To date, 11 species have formally been described in *Kronopolites*, mostly found in China (5 species) and northern Vietnam (2 species). Only a single species each has been reported from northwestern India, northern Thailand, northern Taiwan and northern Laos (Fig. 4). There is little doubt that many more *Kronopolites* species are to be found in the future.

## Supplementary Material

XML Treatment for Kronopolites

XML Treatment for Kronopolites acuminatus

XML Treatment for Kronopolites biagrilectus

XML Treatment for Kronopolites davidiani

XML Treatment for Kronopolites formosanus

XML Treatment for Kronopolites fuscocingulatus

XML Treatment for Kronopolites montanus

XML Treatment for Kronopolites occidentalis

XML Treatment for Kronopolites rugosus

XML Treatment for Kronopolites semirugosus

XML Treatment for Kronopolites swinhoei

XML Treatment for Kronopolites lunatus
